# Loss of Function Mutation in the Palmitoyl-Transferase HHAT Leads to Syndromic 46,XY Disorder of Sex Development by Impeding Hedgehog Protein Palmitoylation and Signaling

**DOI:** 10.1371/journal.pgen.1004340

**Published:** 2014-05-01

**Authors:** Patrick Callier, Pierre Calvel, Armine Matevossian, Periklis Makrythanasis, Pascal Bernard, Hiroshi Kurosaka, Anne Vannier, Christel Thauvin-Robinet, Christelle Borel, Séverine Mazaud-Guittot, Antoine Rolland, Christèle Desdoits-Lethimonier, Michel Guipponi, Céline Zimmermann, Isabelle Stévant, Françoise Kuhne, Béatrice Conne, Federico Santoni, Sandy Lambert, Frederic Huet, Francine Mugneret, Jadwiga Jaruzelska, Laurence Faivre, Dagmar Wilhelm, Bernard Jégou, Paul A. Trainor, Marilyn D. Resh, Stylianos E. Antonarakis, Serge Nef

**Affiliations:** 1 Department of Genetic Medicine and Development, University of Geneva Medical School, Geneva, Switzerland; 2 FHU-TRANSLAD, Département de Génétique, Hôpital Le Bocage, CHU, Dijon, France; 3 EA 4271 GAD Génétique des Anomalies du Développement, Université de Bourgogne, Dijon, France; 4 Cell Biology Program, Memorial Sloan-Kettering Cancer Center, New York, New York, United States of America; 5 Gerstner Sloan-Kettering Graduate School of Biomedical Sciences, Memorial Sloan-Kettering Cancer Center, New York, New York, United States of America; 6 Graduate Program in Pharmacology, Weill Graduate School of Medical Sciences of Cornell University, New York, New York, United States of America; 7 Department of Anatomy and Developmental Biology, Monash University, Clayton, Australia; 8 Stowers Institute for Medical Research, Kansas City, Missouri, United States of America; 9 Institut National de la Santé et de la Recherche Médicale (Inserm) U1085-IRSET, Université de Rennes 1, Structure Fédérative Recherche Biosit, Campus de Beaulieu, Rennes, France; 10 Polish Academy of Sciences, Institute of Human Genetics, Poznań, Poland; 11 EHESP School of Public Health, Rennes, France; 12 Department of Anatomy and Cell Biology, University of Kansas Medical Center, Kansas City, Kansas, United States of America; 13 iGE3, Institute of Genetics and Genomics of Geneva, University of Geneva, Geneva, Switzerland; Max Planck Institute for Molecular Genetics, Germany

## Abstract

The Hedgehog (Hh) family of secreted proteins act as morphogens to control embryonic patterning and development in a variety of organ systems. Post-translational covalent attachment of cholesterol and palmitate to Hh proteins are critical for multimerization and long range signaling potency. However, the biological impact of lipid modifications on Hh ligand distribution and signal reception in humans remains unclear. In the present study, we report a unique case of autosomal recessive syndromic 46,XY Disorder of Sex Development (DSD) with testicular dysgenesis and chondrodysplasia resulting from a homozygous G287V missense mutation in the hedgehog acyl-transferase (*HHAT*) gene. This mutation occurred in the conserved membrane bound *O*-acyltransferase (MBOAT) domain and experimentally disrupted the ability of HHAT to palmitoylate Hh proteins such as DHH and SHH. Consistent with the patient phenotype, HHAT was found to be expressed in the somatic cells of both XX and XY gonads at the time of sex determination, and *Hhat* loss of function in mice recapitulates most of the testicular, skeletal, neuronal and growth defects observed in humans. In the developing testis, HHAT is not required for Sertoli cell commitment but plays a role in proper testis cord formation and the differentiation of fetal Leydig cells. Altogether, these results shed new light on the mechanisms of action of Hh proteins. Furthermore, they provide the first clinical evidence of the essential role played by lipid modification of Hh proteins in human testicular organogenesis and embryonic development.

## Author Summary

Disorders of gonadal development represent a clinically and genetically heterogeneous class of DSD caused by defects in gonadal development and/or a failure of testis/ovarian differentiation. Unfortunately, in many cases the genetic aetiology of DSD is unknown, indicating that our knowledge of the factors mediating sex determination is limited. Using exome sequencing on a case of autosomal recessive syndromic 46,XY DSD with testicular dysgenesis and chondrodysplasia, we found a homozygous missense mutation (G287V) within the coding sequence of the O-acetyl-transferase *HHAT* gene. The *HHAT* gene encodes an enzyme required for the attachment of palmitoyl residues that are critical for multimerization and long range signaling potency of hedgehog secreted proteins. We found that *HHAT* is widely expressed in human organs during fetal development, including testes and ovaries around the time of sex determination. *In vitro* assays show that G287V mutation impairs HHAT palmitoyl-transferase activity and mice lacking functional *Hhat* exhibit testicular dysgenesis as well as other skeletal, neuronal and growth defects that recapitulate most aspects of the syndromic 46,XY DSD patient. These data provide the first clinical evidence of the essential role played by lipid modification of Hedgehog proteins in human testicular organogenesis and embryonic development.

## Introduction

Disorders of sex development (DSD) are rare “congenital conditions in which development of the chromosomal, gonadal or anatomical sex is atypical” [1], and which display a wide spectrum of phenotypes. One clinically and genetically heterogeneous class of DSD is partial or complete 46,XY gonadal dysgenesis [2], caused by a defect in gonadal development and/or a failure of testis differentiation. Individuals with 46,XY complete gonadal dysgenesis (46,XY CGD) are characterized by a 46,XY karyotype, normal female external genitalia, undeveloped (“streak”) gonads, no sperm production, and the presence of Müllerian structures. Despite considerable progress in understanding the genetic factors involved in gonadal differentiation, the causative mutation for individuals with 46,XY CGD remains unknown in 80% of the cases [1], [3], [4]. The majority of resolved cases involve mutations or deletions in genes coding for SRY, desert hedgehog (DHH) , MAP3K1 [5] and NR5A1 (SF1) while the prevalence of duplications involving genes coding for NR0B1 (DAX1) and WNT4 represent ∼1% of the resolved cases [6]. One characteristic of DSD with gonadal dysgenesis is their frequent association with other congenital malformations such as growth or mental retardation, conditions that can be referred to as syndromic DSD [7]. The large variation in cases of syndromic 46,XY DSD involving gonadal dysgenesis suggests that among the network of genes essential for proper development of testes and ovaries, some genes may have pleiotropic actions. The study of syndromic DSD thus provides an opportunity to discover new genes involved in human sex determination and improve the diagnosis and clinical management of DSD patients.

The hedgehog (Hh) family of signaling molecules is composed of three members, namely sonic hedgehog (SHH), desert hedgehog (DHH) and indian hedgehog (IHH). Hh molecules function as morphogens that signal at both short and long range through the patched 1 receptor (PTCH1) in a concentration dependent manner. All Hh ligands are initially synthesized as precursor proteins that undergo auto-proteolytic cleavage and dual lipid post-translational modifications. A cholesterol molecule is attached to the C-terminus of the Hh signaling moiety [8], [9], while the N-terminus is palmitoylated by the membrane-bound O-acyl-transferase hedgehog acyl-transferase (HHAT) [10], [11]. These lipid modifications are required for efficient signaling at both long and short range [12], [13]. In particular, palmitate attachment to SHH is required for the formation of multimeric SHH complexes and increases its signaling potency [10], [12], [14], [15].

Hedgehog ligands play a major role in embryonic patterning, growth and differentiation for a variety of organs and tissues. For instance, SHH acts on neural, cranial and skeletal patterning as well as kidney, lung, tooth and eye development. In the case of bone growth and pancreas development it acts in a synergistic manner with IHH (for review, read [16]). Loss of *SHH* expression or function in humans results in pathologies as diverse as holoprosencephaly [17], preaxial polydactyly [18] or non-syndromic colobomatous microphthalmia [19]. Mutations in *IHH*, on the other hand, more specifically lead to bone growth defects (brachydactyly or acrocapitofemoral dysplasia [20]). Interestingly, several mutations of *DHH* have been described in patients with a non-syndromic form of 46,XY DSD with partial or complete gonadal dysgenesis [2], [21], [22], [23]. Similarly, ablation of *Dhh* in mice leads to severe testis dysgenesis including apolar Sertoli cells, anastomotic testis cords, a reduced number of fetal Leydig cells and insufficient production of androgens, resulting in male infertility, hypogonadism, underdeveloped male accessory glands and feminized external genitalia [24], [25], [26]. In the fetal mouse testis, *Dhh* is expressed specifically in the Sertoli cells from E11.5 onwards [27] and thus is required for testis cord formation, Leydig cell differentiation and possibly germ cell survival [24], [25], [26], [28].

Taking advantage of next generation sequencing technologies, we identified by whole exome sequencing a missense mutation in the palmitoyl transferase gene *HHAT* in a unique case of autosomal recessive chondrodysplasia-46,XY DSD with gonadal dysgenesis (Nivelon-Nivelon-Mabille syndrome). These findings provide the first clinical evidence of the essential role that post-translational lipid modifications have on the functional performance of Hh proteins and hence on human testicular organogenesis and embryonic development.

## Results

### A familial case of syndromic 46,XY complete gonadal dysgenesis with chondrodysplasia

**Figure 1:**
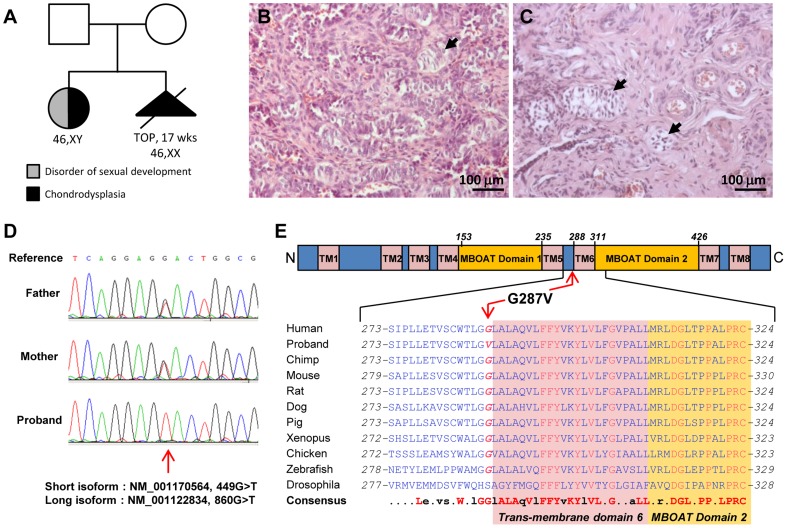
HHAT mutation in 46,XY DSD with chondrodysplasia. A) The familial case presented here consists of two non-consanguineous individuals with two siblings affected with chondrodysplasia. The histological analysis of the dysgenetic gonads revealed the presence of few immature seminiferous tubules in both left (B) and right (C) gonads (arrowheads). D) Sanger sequencing of the proband and parental genomic DNA confirmed the parental origin of the G>T substitution observed at position 860 of HHAT long isoform. E) The mutated Glycine 287 is conserved among vertebrates from zebrafish to humans, and is localized N-terminal to the 6th transmembrane domain of HHAT long isoform.

We studied a family with two children affected by a rare autosomal recessive syndrome associated with DSD and chondrodysplasia (OMIM 600092) [29], [30] (Fig. 1A). The first sibling displayed severe dwarfism with generalized chondrodysplasia, a narrow, bell-shaped thorax, micromelia, brachydactyly, severe microcephaly with cerebellar vermis hypoplasia, facial anomalies, hypoplastic irides, and coloboma of both optic discs. Follow up diagnostic tests at 16 years old confirmed previous clinical observations and identified mild mental retardation, muscular hypertrophy, myopia and other facial anomalies such as upslanting palpebral fissures, puffy eyelids, large mouth, and mild prognathism [30]. The karyotype was 46,XY but the patient exhibited clinical features of a 46,XY DSD with complete gonadal dysgenesis (CGD), including normal external female genitalia, lack of pubertal development, primary amenorrhea, and hypergonadotrophic hypogonadism. Histology confirmed the testicular dysgenesis and identified persistent Müllerian and Wolffian duct structures, in particular epididymal remnants. The left gonad contained large inclusions of immature testicular tissue mainly composed of dysplastic immature seminiferous tubules (Fig. 1C - arrowhead). These structures were delimited with a dense interstitial compartment containing a greatly reduced proportion of cells expressing the cholesterol side-chain cleavage enzyme CYP11A1 compared to normal adult testis (Fig. S1). The right gonad was hypoplastic and largely composed of fibroblastic and endothelial tissues. It contained some structures that resembled immature seminiferous tubules surrounded by myoid cells (Fig. 1D - arrowheads). No CYP11A1-expressing cells were detected around these structures (data not shown). It is possible that a small group of functional steroidogenic cells may have developed and persisted in the dysgenic gonads of the patient, in a number sufficient to explain the presence of vestigial Wolffian duct derivatives, however insufficient to achieve complete and normal development of the male genitalia.

The second sibling had a 46,XX karyotype with histologically normal ovaries, but otherwise exhibited similar phenotypic abnormalities including severe dwarfism and generalized chondrodysplasia [29]. Pregnancy was interrupted after ultrasound examination at 17 weeks of gestation. To date no other similar case has been reported.

### Homozygous G287V missense mutation in the *HHAT* gene as a cause for Nivelon-Nivelon-Mabille syndrome

A 244K aCGH analysis performed on genomic DNA extracted from blood cells of the patient did not reveal any chromosomal rearrangements that could account for the pathology. All the copy number variations (CNV) identified during the analysis were annotated in the database of genomic variants (DGV) as benign (data not shown). We then performed targeted exon capture and next-generation sequencing on DNA from the 46,XY DSD proband and her parents (Table 1). Variants were evaluated using VariantMaster [31], for *de novo*, X-linked and recessive models (Table 2).

**Table 1:** Number of reads, coverage and variants identified per individual.

	father	mother	patient
Total reads	174'432'036	204'738'642	200'725'004
Reads after removing duplicates	99'626'106	109'452'796	109'029'488
On target*	60'463'056	65'192'523	64'962'546
Mean coverage*	116.5	124.3	124.7
Coverage ×8*	93.8%	93.9%	94.6%
Coverage ×20*	87.1%	87.5%	88.6%
Synonymous SNV	10'446	10'635	10'648
Missense SNV	9'596	9'607	9'724
Total exonic	21'604	21'922	22'032
Splicing (±10 bp)	3'955	4'046	4'107
Total exonic + splicing	25'559	25'968	26'139

SNV: Single Nucleotide Variant.

*The target is the total protein coding sequence of the human genes according to RefSeq.

**Table 2:** List of variants identified for the different models tested.

Chr	Position	Gene	NCBI	cDNA	Protein change	dbSNP	MAF	SIFT/NNSplice*	PolyPhen2/ESEfinder*	Mutation Taster*	GERP++
**De novo model**											
chr1	1.1E+08	CELSR2	NM_001408.2	c.50T>C	p.(Leu17Pro)	rs200277265	NA	0	0	0	−0.926
**X-linked model**											
chrX	10104754	WWC3	NM_015691.3	c.2845C>T	p.(Arg949Cys)	rs56399961	0.002226	0.013	0.999	0.9999	5.4
chrX	1.29E+08	XPNPEP2	NM_003399.5	c.644C>T	p.(Thr215Ile)	rs138365897	0.001908	0.004	0.838	0.9958	3.66
chrX	1.54E+08	F8	NM_000132.3	c.1272-5T>C	NA	NA	NA	0.9>0.8	9.3>8.9	NA	−4.07
**Recessive model**											
chr1	2.11E+08	HHAT	NM_001170564.1	c.449G>T	p.(Gly150Val)	NA	NA	0.001	1	0.9988	4.98

MAF: Minimum Allele Frequency.

*Scores for the different pathogenicity prediction scores. For the splice variant NNSplice and ESEfinder were used instead of SIFT, PolyPhen2 and Mutation Taster.

One *de novo* variant was identified in *CELSR2* (NM_001408.2:c.50T>C:p.(Leu17Pro), OMIM: 604265) which is predicted as non-pathogenic by PolyPhen2 and Mutation Taster, and pathogenic by SIFT. RNAi-based knockouts of *Celsr2* in mice resulted in prominent simplification of the dendritic arbors of cortical pyramidal neurons and Purkinje neurons [32], which in combination with the low pathogenicity score led us to not consider this variant further. Among the X-linked variants the NM_000132.3:c.1272-5T>C in *F8* (OMIM: 300841) was not considered, as both programs (i.e. NNSplice and ESEfinder) predicted a slight decrease of the acceptor splice site, which we did not consider as potentially pathogenic. No data were found in the literature that allowed us to evaluate the potential role of WWC3 in the patients' phenotype. *XPNPEP2* (OMIM: 300145) has been implicated in ACE inhibitor-induced angioedema [33]. The rather high allelic frequency for both *WWC3* and *XPNPEP2* variants (∼1/2000) coupled with the absence of a clear functional link between these genes and the patient phenotype led us to consider them as non-pathogenic or at least non-related to the patient's phenotype.

The NM_001170564.1:c.449G>T:p.(Gly150Val) in the *HHAT* short isoform, equivalent to NM_001122834:c.860G>T:p.(Gly287Val) in the *HHAT* long isoform (OMIM: 605743), was the only variant remaining after filtering variants for the recessive model. The variant is homozygous in the patient and heterozygous in the parents. Inheritance of the newly identified mutation was confirmed by Sanger sequencing (Fig. 1D). All pathogenicity prediction algorithms, i.e. SIFT, PolyPhen and Mutation Taster, consider this mutation as deleterious, damaging or pathogenic (Table 2). G287V is located just next to the 6th predicted transmembrane domain and in the membrane bound *O*-acyltransferase (MBOAT) domain, two highly conserved regions of HHAT (Fig. 1E). The G287 residue is also well conserved (GERP++: 4.98) across species from zebrafish to mice and humans (Fig. 1E), suggesting an important role for this amino-acid in either HHAT protein folding, stability or the acyltransferase activity required for palmitoylation of Hh proteins. In addition, the pleiotropic phenotype including dwarfism, chondrodysplasia, brachydactyly and gonadal dysgenesis is consistent with previously described defects in SHH, DHH and IHH signaling [34].

### *HHAT* is widely expressed in mouse and fetal human organs including ovaries and Sertoli cells in developing testes

**Figure 2:**
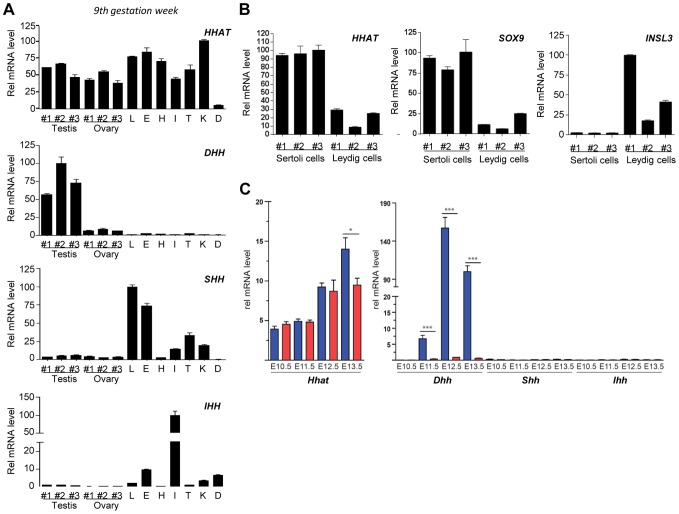
HHAT is widely expressed during human fetal development and post-natal life. A) qRT-PCR analysis of *HHAT* and Hedgehog protein coding genes (*DHH*, *IHH* and *SHH*) expression in human fetal tissues at Gestation Week 9. B) qRT-PCR analysis reveals that HHAT is preferentially expressed in human Sertoli cells (SOX9-expressing cells) compared to Leydig cells (INSL3-expressing cells). C) Evaluation of the expression of *Hhat* (left panel) and Hedgehog genes (right panel) by qRT-PCR in mouse SF1^+^ somatic cells from developing XY (blue bars) and XX (red bars) gonads. Legend: L, lung; H, Heart; I, Intestine; T, Tongue; K, Kidney; D, anterior limb digits.

In order to obtain insight into the potential role of HHAT during fetal development, we characterized its expression in several human tissues, including fetal testes and ovaries, just after the time of sex determination (gestation week (GW) 9). Expression analysis revealed that *HHAT* is expressed at various levels in most of the fetal human organs investigated, including testes and ovaries (Fig. 2A). Interestingly, the different hedgehog genes showed more specific expression profiles. As expected, *DHH* is specifically expressed in the embryonic testis, whereas strong *SHH* expression is detected in the fetal lung and eye and is absent from the fetal heart at this developmental stage (Fig. 2A). We observed a strong expression of *IHH* in the small intestine and digits, consistent with its well-established role in the development of these structures [35], [36]. In the adult testis, qRT-PCR from purified human adult Sertoli cells, expressing *SOX9*, and Leydig cells, expressing *INSL3*, indicated that *HHAT* is expressed at high levels in Sertoli cells, the unique gonadal source of Hh ligands [27], [37] (Fig. 2B). In mice, transcriptional analysis of XX and XY SF1-positive gonadal cells during sex determination confirmed that *Dhh* is strongly and specifically expressed in XY cells starting at E11.5 (Fig. 2C), a profile consistent with its reported expression in Sertoli cells [27]. In contrast, *Hhat* transcripts are already present in the somatic progenitors of both XX and XY gonads at E10.5 prior to sex determination. Following testicular differentiation, there is a 2.5-fold increase in *Hhat* transcripts levels in XY SF1^+^ cells by E13.5 (Fig. 2C). Overall, the broad expression of *HHAT* during development is consistent with the wide-ranging functions of Hh in mediating organogenesis and embryonic patterning. In gonads, our expression analysis suggests that *HHAT* is expressed in the supporting cell lineage at the time of sex determination and later in Sertoli cells.

### G287V mutation impairs HHAT palmitoyl-transferase activity *in vitro*

**Figure 3:**
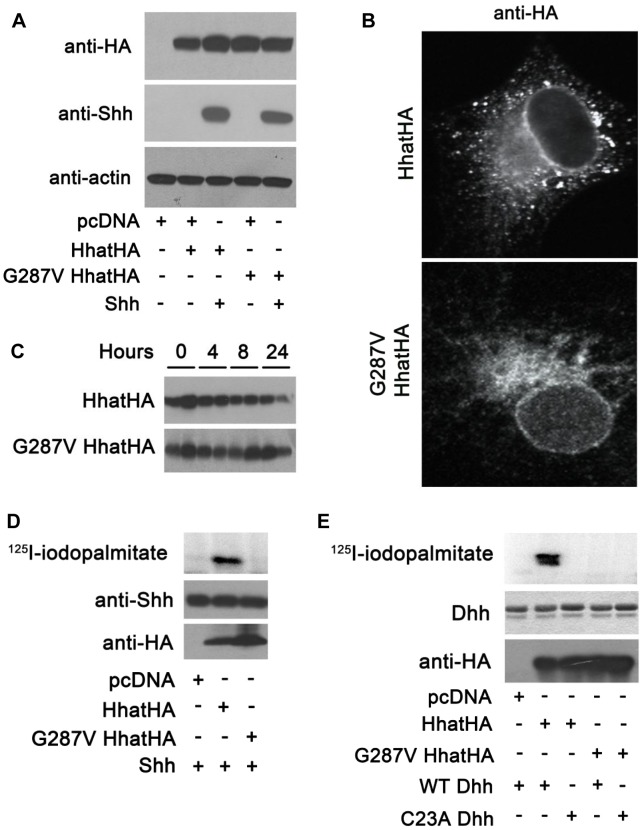
G287V mutation results in loss of HHAT activity. A) COS-1 cells were transfected with the indicated constructs and cell lysates were analyzed directly by Western blotting. B) COS-1 cells transfected with wild type or G287V HHAT-HA were fixed and processed for indirect immunofluoresence and stained with the antibodies indicated. C) COS-1 cells were transfected with the indicated HHAT construct and incubated in DMEM supplemented with 10% FBS, 100 µg/ml cyclohexamide, and 40 µg/ml chloramphenicol. At each indicated time point, cells were lysed and subjected to SDS-PAGE and Western blotting with anti-HA antibodies. The amount of HA signal at each time point was determined using GS-800 Calibrated Densitometer (BioRad). Experiments were carried out in duplicate and repeated three times. D) *In vitro* palmitoylation assay using SHH and membranes from cells expressing wild type or G287V HHAT. Top panel, incorporation of ^125^I-iodopalmitate into SHH detected by phosphorimaging. Middle panel, Anti-SHH Western blot. Lower panel, Anti-HA Western blot. Experiments were carried out in duplicate and repeated three times. E) *In vitro* palmitoylation assay using DHH and membranes from cells expressing wild type or G287V HHAT. Top panel, incorporation of ^125^I-iodopalmitate into DHH detected by phosphorimaging. Middle panel, Coomassie-staining of DHH. Lower panel, Anti-HA Western blot. Experiments were carried out in duplicate and repeated three times.

To further investigate the effect of the G287V mutation on HHAT function, plasmids encoding wild type and G287V HA-tagged HHAT were generated and expressed in COS-1 cells. Both wild type and G287V HHAT were expressed at equivalent levels in the presence or absence of co-transfected SHH, which in turn was expressed at the same level in the presence of either wild type or mutant HHAT (Fig. 3A). Immunofluorescence staining revealed that the subcellular localization of wild type and G287V HHAT was similar; both proteins localized in the endoplasmic reticulum (ER) and the Golgi (Fig. 3B). To test whether the G287V mutation altered protein stability, cells expressing wild type or mutant HHAT were treated with cycloheximide and chloramphenicol, and the amount of HHAT protein remaining at various time points was measured. The stability of the G287V mutant was comparable to that of wild type HHAT (Fig. 3C).

We next used an *in vitro* assay for HHAT activity, which quantifies the incorporation of ^125^I-iodopalmitate into Hh proteins, to compare the activity of G287V HHAT to that of wild type. Membranes from cells expressing either wild type or G287V HHAT were incubated with ^125^I-iodo palmitoyl CoA and purified, recombinant SHH protein. In the presence of wild type HHAT, robust incorporation of ^125^I-iodopalmitate into SHH was detected (Fig. 3D). By contrast, no incorporation of ^125^I-iodopalmitate into SHH was detected with G287V HHAT. DHH is the only hedgehog protein expressed in gonads and is likely the more relevant Hh family substrate in this system. To date, no study has tested the ability of HHAT to palmitoylate DHH. We purified a recombinant DHH protein encompassing the N-terminal signaling fragment, along with a C23A DHH mutant, which lacks the N-terminal Cys residue required for palmitoylation. Using the *in vitro* HHAT activity assay, we provide the first evidence that HHAT directly palmitoylates DHH and that the G287V mutation results in loss of activity (Fig. 3E). Taken together, these results clearly indicate that G287V HHAT lacks the ability to palmitoylate hedgehog proteins.

### Palmitoylation of hedgehog ligands is required for testis organogenesis in mice

In mice, *Hhat* loss of function recapitulates many of the skeletal and growth defects observed in the patients described above, as well as in human individuals bearing mutations in genes encoding Hh proteins [34]. Mice lacking functional HHAT developed to term but died soon after birth. Mutant mice displayed severe dwarfism, short-limbs, diminished chondrogenesis and osteogenesis, facial defects, and holoprosencephaly together with acrania and agnathia [12], [38]. Since defects in testicular differentiation and development have never been investigated in these mutant mice, we analyzed by histological means and immunofluorescence the pattern of expression of key somatic and germ cell markers in the developing gonads of wild type (WT) and *Hhat^Creface/Creface^* mutant (Creface) mice [38]. The developing XX mutant gonads (E11.5–E15.5) were histologically indistinguishable from XX control gonads (Fig. S2A–H). As expected, none of the testis-specific markers including SOX9 and CYP11A1 were expressed (data not shown), but cells positive for the key ovarian-promoting factors FOXL2 were normally present in XX mutant gonads at E13.5 (Fig. S2J). As expected, female germ cells were normal in numbers and entered meiosis at around E13.5, as shown by the expression of MVH and γH2AX, which were similar in both control and mutant XX gonads (Fig. S2M,N & O,P). Thus, we conclude that ovarian differentiation occured normally in XX mutant gonads.

**Figure 4:**
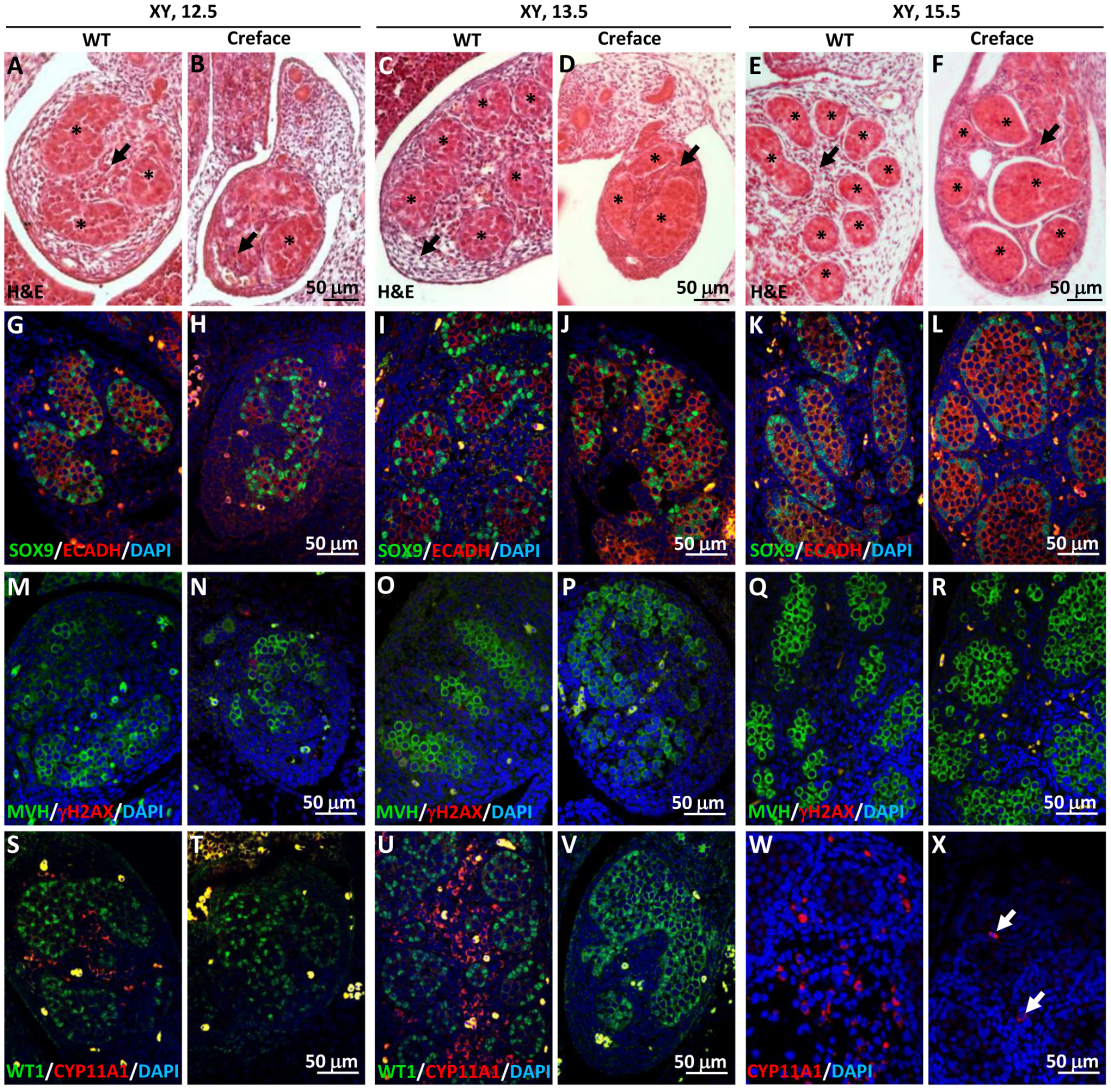
Testicular dysgenesis in mouse embryo lacking *Hhat*. A–F) H&E staining of wild-type (WT) and *Hhat^Creface/Creface^* mutant (Creface) XY gonads at E12.5 (A, B), E13.5 (C, D) and E15.5 (E, F). Note the reduction in testis size from E12.5 onward and the presence of dysgenetic testis cords (asterisk) with considerable variations in term of shape and size. Finally, the interstitial space (arrowhead) in the E13.5 and E15.5 mutant testes was abnormally dense and cellular in comparison of control mice testis. G–X) Expression of key testicular markers was assessed by double/single immunofluorescence at E12.5, E13.5 and E15.5 on both wild-type (WT) and *Hhat^Creface/Creface^* mutant (Creface) XY gonads using either the Sertoli cell marker SOX9 (green) and the germ cell marker ECADH (red, G–L), the meiotic marker γH2AX (red) along with the germ cell marker MVH (green; M–R) or the Sertoli cell marker WT1 (green) with the Leydig cell marker CYP11A1 (red, S–X). Note the near complete absence of Leydig cells in mutant testes.

In XY embryos, no obvious differences were observed between mutant and control XY gonads at E11.5 (Fig. S3A, B). SRY-positive pre-Sertoli cells were present (Fig. S3C, D), indicating that the testicular program was normally initiated, despite the absence of functional HHAT. However, our analysis revealed that testis development was severely affected at subsequent stages, leading ultimately to testicular dysgenesis. We observed a drastic reduction in testis size, which was apparent from E12.5 to E15.5 (Fig. 4A–F). In addition, H&E staining (Fig. 4A–F) as well as immunofluorescence for marker proteins of germ cells (ECADH and MVH) and Sertoli cells (SOX9 and WT1) (Fig. 4G–V), demonstrated not only a decrease in testis cord numbers between wild-type and mutant testes, but also an alteration of the size and shape of testis cords at all stages investigate, which appeared irregular and anastomotic (Fig. 4). Interestingly, while testis cord formation was affected, the differentiation of Sertoli cells appeared to be normal. Both, the expression of SOX9 and WT1, two Sertoli cells markers, were present in XY mutant gonads at all stages investigated (Fig. 4G–L & S–V). These data collectively suggest that HHAT is not required for Sertoli cell commitment but plays a role in proper testis cord formation.

The interstitial compartment of the developing mutant testes at E13.5 and E15.5 exhibited a high cellular density typical of irregular and dense connective tissues, in sharp contrast with the loose structure generally observed in control testes at these developmental ages (compare arrows in Fig. 4 D&F with C&E). In fact, we found that the differentiation of fetal Leydig cells and steroidogenesis were not initiated in mutant gonads, indicated by the absence of the Leydig cell-specific marker CYP11A1 at E12.5 and E13.5 (Fig. 4 T&V). Nevertheless, few rare CYP11A1-positive cells were observed at E15.5 (arrows in Fig. 4X), an expression pattern reminiscent to what was found in the patient gonads (Fig. S1D). Overall, the analysis of the *Hhat^Creface/Creface^* mutant XY gonads confirmed that the loss of functional HHAT affects the development of fetal Leydig cells as well as the proper formation of testis cords, leading ultimately to testicular dysgenesis.

## Discussion

We used whole-exome sequencing (WES) for the identification of the underlying genetic cause of a unique case of autosomal recessive syndromic 46,XY DSD with testicular dysgenesis and chondrodysplasia. Analysis of WES results using an autosomal recessive model revealed a homozygous G287V missense mutation in the hedgehog acyl-transferase (*HHAT*) gene. This gene encodes an endoplasmic reticulum protein, HHAT, which catalyzes the transfer of palmitate onto hedgehog ligands. The Gly287 residue is a highly conserved amino acid which, when mutated, leads to a non-functional HHAT protein that lacks the ability to palmitoylate hedgehog proteins. Consistent with the patient phenotype, HHAT is expressed in a wide variety of human fetal organs as well as in Sertoli cells, the unique source of DHH in the testis. In addition, *Hhat* loss-of-function in mice recapitulates most of the testicular, skeletal, neuronal and growth defects observed in the patient. Overall, these data emphasize the essential role played by post-translational lipid modification of Hh ligands in mediating patterning of many aspects of the body, including the testis, limbs, axial skeleton and the central nervous system.

### Role of Hedgehog palmitoylation in embryonic development

The patient familial history indicates that the patient and sibling were conceived by a healthy, non-consanguineous caucasian couple [29]. Despite an extensive search through large cohorts of patients suffering from skeletal disorders and/or DSD, we have not been able to identify a second familial case with a pathogenic mutation in the *HHAT* locus. This suggests that the frequency of pathogenic variants of *HHAT* is extremely low within the general population, which would explain the absence of other listed cases of Nivelon-Nivelon-Mabille syndrome. It is possible that other mutations in the *HHAT* locus may lead to severe defects in HHAT cellular localization, expression and/or activity that might cause lethality at the embryonic or fetal stages. Alternatively, additional variants may be found in the non-coding regulatory regions of the gene, as is the case for SHH and other genes [39], [40].

The wide range of developmental and testicular anomalies observed in both the patient and mice lacking the capacity to palmitoylate Hh proteins is consistent with a perturbation of SHH, DHH and IHH signaling. When tested *in vivo* or with *in vitro* cell-based assays, palmitoylation of SHH has been shown to be required for the formation of soluble multimeric complexes which enhances its signaling potency [10], [12], [14], [15]. In agreement, mice with *Hhat* loss-of-function fail to properly establish a long-range Hh signaling gradient [38] which results in testicular, neuronal, skeletal and limb patterning defects [12], [38] similar to those observed in *Shh* [41], [42], *Dhh* [24], [25], [26] and *Ihh* [35], [43] knockout mice. Interestingly, mouse embryos expressing a non-palmitoylated form of SHH (*Shh^C25S^*) exhibit similar defects to that of *Hhat* mutant embryos due to defective SHH signaling [12]. These similar phenotypes indicate that Hh proteins are indeed the major targets of HHAT.

Surprisingly, the clinical characteristics and defects observed in the patient bearing the G287V mutation are not as severe as the phenotypes found in *Shh^C25S^* mutant or *Hhat* knockout mice [12], [38]. While homozygous *Hhat* mutant mice die around birth, the patient exhibits normal viability despite severe skeletal, craniofacial and neuronal defects. This could be explained by species differences and/or a small residual transferase activity in the G287V-HHAT mutant protein that is not detected in our *in vitro* assays.

The role of Hh proteins in regulating embryonic pattern formation and adult tissue maintenance in mammals and the deleterious effects that accompany their ablation has been extensively studied (for review see [16]). In the present study, we face a novel and peculiar situation where Hh primary sequences are not affected, but rather the establishment of morphogen gradients and subsequent long range signaling actions of all three Hh ligands. This is reflected by the variable severity of the multiple Hh gene-related symptoms described in this patient. For instance, the impaired testicular development suffered by the patient reaches a severity that is close, if not identical, to what was observed in previous cases of DHH mutation [2], [21], with the generation of streak gonads that do not contain functional reproductive structures. Similarly, the patient suffered a severe form of type A1 brachydactyly, which affects each of the five fingers – a situation typically observed in patients with IHH mutations [44]. In these particular cases, the severity of the symptoms might reflect the necessity to establish a proper long-range Hh gradient for gonadal organogenesis and finger development. On the other hand, the relatively mild abnormalities observed in optical disc and the absence of holoprosencephaly in the patient are in stark contrast with the severe craniofacial defect and abnormal forebrain development found in patients with SHH loss of function [17], [45]. These differences in severity might account for the fact that forebrain development into two separated hemispheres does not rely on the establishment of a long range SHH gradient as much as other patterning SHH-related processes.

### Lipid modifications of Hh ligands are conserved during evolution

The core components required for Hh production, movement and signal transduction events are conserved between *Drosophila* and humans [46], [47]. This is particularly true for the mechanisms of post-translational dual lipid modification of Hh ligands [9], [10]. This is required to generate soluble, multimeric forms of Hh, create the Hh-signaling gradient and to enhance Hh-signaling potency [13]. Palmitoylation is one of the two critical lipid modifications [48] and is mediated by the hedgehog HHAT. This protein is highly conserved, particularly in transmembrane regions and MBOAT domains. It is noteworthy that the mutated G287 residue, one of the highly conserved residues in vertebrate evolution, is positioned adjacent to the predicted 6^th^ transmembrane region and 2^nd^ MBOAT domain (Fig. 1E). Importantly, the G287V mutation does not affect the subcellular localisation and stability of mutant HHAT, both of which are comparable to that of wild type HHAT. It does, however, impair its ability to palmitoylate hedgehog ligands. Further work will be required to define the precise structural role of G287 in mediating HHAT protein folding and its palmitoyl-transferase activity.

### HHAT is essential for testicular development and fetal Leydig cell differentiation

Our expression studies using human and mouse embryonic tissues as well as purified cells indicate that *HHAT* is expressed in developing gonads around the time of sex determination, probably in the supporting cell lineage, and later in Sertoli cells, which are the sole source of hedgehog ligand in the gonad [27], [37]. The absence of functional HHAT leads to testicular dysgenesis both in humans and in the knockout mouse model. Careful analysis of testis development during the sex determination period revealed that early stages of gonad development occur normally up to E11.5 in *Hhat^Creface/Creface^* mice, and that the initiation of testicular program is not affected. However, by E12.5 testicular development was significantly impaired in XY embryos with a massive reduction in testis size, a reduction in the number of testis cords that also appeared irregular in shape and diameter, and a almost complete absence of fetal Leydig cells. Thus HHAT is not required for Sertoli cell commitment but plays a role in proper testis cord formation and the differentiation of Leydig cells. In this context, it is interesting to note that *Hhat^Creface/Creface^* testicular phenotype was similar to the one observed in *Dhh* knockout mice. Knockout mice for the *Dhh* gene display testis dysgenesis including apolar Sertoli cells, anastomotic testis cords, a decreased number of fetal Leydig cells and an insufficient production of androgens resulting in male infertility, hypogonadism and feminized external genitalia [24], [25], [26]. Consistent with its role in the mouse, several mutations of *DHH* have also been described in patients with a non-syndromic form of 46,XY DSD with partial or complete gonadal dysgenesis [2], [21], [22], [23]. Although it is now clear that HHAT is essential for testis formation in both humans and mice, further work will be required to define the precise role of HHAT and Hh palmitoylation in mediating testis development and how it influences testis cord formation and the specification of fetal Leydig and peritubular myoid cells.

## Materials and Methods

### Patient management, sample collection and DNA extraction

All clinical investigations have been performed according to Declaration of Helsinki principles. The study was approved by the local French ethics committee (#DC2011-1332). The proposita was described in [29], [30] and referred at 16 years of age for follow-up. The patient was seen at the Medical Genetics Center of Dijon in September 2004. She presented mild mental retardation, a dysmorphic face, an anteriorly bent thorax with a rigid spine, brachydactyly, muscular hypertrophy and myopia. Endocrine studies performed because of lack of pubertal development showed hypergonadotrophic hypogonadism. Gonadectomy was performed in order to prevent malignant transformation. The proband's parents had a normal phenotype. Genomic DNA from the proband and her parents were isolated from blood samples using the Qiagen DNA mini kit (Qiagen, Valencia, California). An array-CGH 244K (Agilent) has excluded pathogenic microdeletions and microduplications.

### Exome capture and sequencing

Exome capture was performed using the SureSelect Human All Exon v3 kit (Agilent Inc). Sequencing was carried out on an Illumina HiSeq 2000 instrument. Fastq files were obtained using the Illumina CASAVA v1.8.1 software and processed using our “in house” bioinformatic pipeline running on the Vital-IT Center for high-performance computing of the Swiss Institute of Bioinformatics (SIB; http://www.vital-it.ch). This pipeline utilizes published algorithms in a sequential manner (BWA [49] for map reads, SAMtools [50] for detection of variants, Pindel [51] for the detection of indels, ANNOVAR [52] for the annotation). The entire coding sequence corresponding to the RefSeq [53] coding genes was used as the reference for the calculation of coverage and reads on target. All experiments were performed using the manufacturer's recommended protocols without modifications.

Results were analyzed using the VariantMaster software [31] in order to identify *de novo* variants as well as variants respecting different Mendelian inheritance models (dominant with reduced penetrance, recessive, X-linked). Variant validation was performed by targeted Sanger sequencing.

### Human tissue collection and Sertoli and Leydig cell purification

Human fetal gonads (9 gestational weeks) were obtained from pregnant women referred to the Rennes University Hospital (France) for legal abortion in the first trimester as previously described [54]. All women received information and gave verbal consent in accordance with national guidelines (Agence de la Biomédecine, authorization #PFS09-011) and protocols were approved by the local ethics committee of Rennes (#11-48). The termination of pregnancy was induced by treatment with Mifegyne (mifepristone) followed 48 h later by Cytotec treatment, and finalized by aspiration. None of the terminations were motivated by fetal abnormality. After recovery from the aspiration product under a binocular microscope (Olympus SZX7, Lille, France), gonads and other somatic organs were snap frozen and stored at −80°C until RNA extraction.

Adult human testes were collected either from prostate cancer patients undergoing orchiectomy who had not received any hormone therapy or from brain dead men with the authorization from the Agence de la Biomédecine (authorization # PFS09-015, “Etude de la spermatogenèse humaine normale et pathologique”). Leydig cells were isolated using a combination of mechanical dissociation, enzymatic digestion, filtration through nylon meshes and density Percoll gradient centrifugation, as previously described [55]. Human primary Sertoli cells were purchased from Lonza (Basel, Switzerland) following isolation procedures described elsewhere [56], [57].

### Isolation of mouse Sf1:GFP positive cells and RNA extraction

Urogenital ridges from individual embryos bearing a Sf1-BAC-eGFP transgene-positive urogenital ridges [58], [59] were dissected at relevant stages (E10.5, E11.5, E12.5 and E13.5), digested with trypsin/EDTA, filtered through a 40-mm cell strainer to generate single-cell suspensions and then sorted using a FACS Vantage SE with a purity of above 97% as previously described [58].

### Quantitative Reverse Transcriptase PCR (qRT-PCR)

Total RNA from human samples was isolated using Macherey Nagel RNA isolation kit, and 250 ng of total RNA were DNase-treated and converted to 1^st^ strand cDNA using SuperScript II Reverse Transcriptase following the manufacturer's instructions (Invitrogen Life Technologies). RNA from Sf1^+^ cells was extracted using RNeasy microkit from Qiagen according to the manufacturer's protocol and amplified RNAs were obtained and converted into double-stranded cDNA as previously described [58]. qPCR was carried out in optical 100-well plates and labeled by using the SYBR green master mix (Applied Biosystems). The fluorescence was quantified with a Prism 7900 HT sequence detection system (Applied Biosystems). The expression of each gene was assayed in triplicate as previously described [60], with mouse *Gapdh* and human *RPL19* genes used as reference genes. Primers used for qRT-PCR are listed in Table S1 and were designed using the software PRIMER EXPRESS (Applied Biosystems). The statistical significance of fold-changes was determined by a paired Student's *t*-test.

### Reagents and antibodies

Coenzyme A, CoA synthetase, and anti-HA antibodies were purchased from Sigma (St. Louis, MO). Anti-SHH and anti-CYP11A1 antibodies were purchased from Santa Cruz Biotechnology. Anti-γH2AX, anti-MVH and anti-ECADH antibodies were purchased from Cell Signaling Technology, R&D Systems and Becton Dickinson (USA), respectively. Anti-WT1 was purchased from Dakocytomation (CA, USA). Anti-SRY, anti-SOX9, anti-FOXL2 antibody were previously described [61]. Alexa-conjugated secondary antibodies were purchased from Invitrogen. [^125^I] NaI was obtained from Perkin Elmer.

### Mammalian expression plasmids

The full length *SHH* and the wild type *HHAT* cDNA plasmids, in a pcDNA3.1 backbone with a C-terminal HA-tag, have been previously described [11]. The G287V *HHAT* construct was generated from wild-type *HHAT* by site directed mutagenesis using the QuikChange mutagenesis kit (Stratagene). A plasmid encoding full length *DHH* was purchased from Origene. To generate the *DHH* (23–198) construct for purification, an *Nde*I restriction site was introduced preceding basepair 69 of DHH. A *Bam*HI site followed by a stop codon was introduced preceding basepair 597. The digested fragment was ligated into *Nde*I and *Bam*HI digested pET19b (Novagen). The CATATG sequence at the beginning of *DHH* was deleted using QuikChange to generate the N-terminally His6-tagged human *DHH* (23–198), with an enterokinase cleavage site immediately preceding residue 23 of *DHH*. The C23A *DHH* construct was generated by site directed mutagenesis using QuikChange. All constructs and mutations were confirmed by DNA sequencing.

### Cell culture and transfection

COS-1 cells were grown in Dulbecco's modified Eagle's medium supplemented with 10% fetal bovine serum, 50 units/ml penicillin, and 50 µg/ml streptomycin. 293FT cells were grown in Dulbecco's modified Eagle's medium supplemented with 10% fetal bovine serum, 50 units/ml penicillin, 50 µg/ml streptomycin, 500 µg/ml Geneticin, 1 mM GlutaMAX (Invitrogen), 1 mM sodium pyruvate, and 0.1 mM nonessential amino acids. Transfections were carried out using Lipofectamine 2000 (Invitrogen).

### Expression of purified recombinant DHH

His-tagged SHH, DHH (23–198) and C23A DHH (23–198) were expressed in *Escherichia coli* BL21(DE3)pLysS cells, purified on Ni-NTA-agarose resin, and dialyzed (20 mM Tris-HCL pH8.0, 350 mM NaCl, and 1 mM β-mercaptoethanol) in the presence of 6 ng/mL enterokinase. The protein concentration was measured using the DC protein assay (Bio-Rad). The N termini of both wild-type and mutant proteins were confirmed by Edman degradation.

### *In vitro* palmitoylation assay

293FT cells were transfected with plasmids encoding wild type or G287V HHAT. Cells were lysed in hypotonic lysis buffer (0.2 mM MgCl_2_ and 10 mM HEPES, pH 7.3) 48 hours post transfection. After Dounce homogenization, sucrose was added to a final concentration of 0.25 M and the membrane fraction was pelleted by ultracentrifugation at 100,000 g for 45 min in a Beckman Ti-70.1 fixed angle rotor. The pellet was resuspended in the hypotonic lysis buffer containing 0.25 M sucrose and flash frozen. 10 µg of membranes were incubated with 10 µl of recombinant SHH or DHH (0.2 mg/ml in 20 mM MES (pH 6.5), 1 mM EDTA, and 1 mM DTT), followed by the addition of 30 µl of reaction buffer (167 mM MES (pH 6.5), 1.7 mM DTT, 0.083% Triton X-100, and 167 µM ^125^I-iodopalmitoylCoA (synthesized using CoA synthetase as previously described [62]). After one hour, the reaction was stopped by the addition of 50 µl of 2× sample buffer with 40 mM DTT. Samples were electrophoresed on SDS-PAGE gels, dried, and exposed to phosphorimaging for 12–18 h.

### Protein stability assay

COS-1 cells were transfected with wild type or G287V *HHAT*. 48 hours post-transfection, cells were incubated in media supplemented with 100 µg/ml cyclohexamide and 40 µg/ml chloramphenicol for 0, 4, 8, 10, or 24 hours. Cells were lysed in 500 µl radioimmunoprecipitation assay (RIPA) buffer (150 mM NaCl, 50 mM Tris (pH 7.4), 1% Triton X-100, 0.5% sodium deoxycholate, 0.1% SDS, and 1 mM EDTA), electrophoresed on SDS-PAGE gels, transferred to PVDF membranes and probed with anti-HA antibody to determine protein levels.

### *Hhat* knockout mouse line

The *Hhat+/Creface* mouse line was established and maintained by Paul A. Trainor at the Stowers Institute for Medical Research. These mice were housed in the Laboratory Animal Services Facility at the Stowers Institute for Medical Research according to IACUC animal welfare guidelines. *Hhat^Creface/Creface^* embryos and control littermates were generated and genotyped by classic PCR as described previously [38]. Embryos were collected from timed matings and staged by designating noon of the day on which the mating plug was detected as E0.5. Embryos were fixed overnight in 4% paraformaldehyde (PFA) and embedded in paraffin. Sections (5-µm) were stained with hematoxylin and eosin (H&E) or processed for immunofluorescence (IF).

### Histology and immunofluorescence

Cells were transfected with wild type or G827V *HHAT* and grown on coverslips for 24 hours. Cells on coverslips were fixed in 4% paraformaldehyde, permeabilized with 0.2% TritonX-100, and stained with indicated antibodies and Hoechst dye. Coverslips were mounted on slides using ProLong Gold mounting solution (Invitrogen). Images were collected on a Zeiss LSM 510 microscope using a 63× water immersion objective.

The patient gonads and healthy human adult testes were fixed in formol whereas mouse samples used for immunofluorescence were fixed in paraformaldehyde. Five µm-sections were cut from paraffin embedded samples and stained with hematoxylin and eosin (H&E), or processed for IF and stained with indicated antibodies and DAPI. All images were obtained with a Zeiss Axioscop or a Nikon C1 Upright microscope.

## Supporting Information

Figure S1 CYP11A1-expressing cells are reduced in the dysgenetic XY gonads of the patient with *HHAT* mutation. Haematoxylin and eosin staining (A,B) as well as immunostaining for the Leydig markers CYP11A1 (C,D) from testis of a fertile adult men (A,C) and the left gonads of the patient bearing G287V mutation in HHAT (B, D).
(PDF)

Figure S2 Ovarian differentiation is normal in XX mice embryos lacking *Hhat*. A–H) H&E stained sections of wild-type (WT) and *Hhat^Creface/Creface^* mutant (Creface) XX gonads at E11.5 (A, B), E12.5 (C, D), E13.5 (E, F) and E15.5 (G, H). I–P) Expression of key ovarian markers was assessed by double immunofluorescence at E12.5, E13.5 and E15.5 on both wild-type (WT) and *Hhat^Creface/Creface^* mutant (Creface) XX gonads using either FOXL2 (green) and the germ cell marker ECADH (red, I–J) or the meiotic marker γH2AX (red) along with the germ cell marker MVH (green; K–P). Dotted lines mark the developing gonads at E11.5 (A,B).
(PDF)

Figure S3 SRY is expressed in the genital ridges of XY embryos lacking *Hhat* gene. Haematoxylin and eosin staining (A,B) as well as immunostaining for SRY (C,D) from WT (A–C) and *Hhat^Creface/Creface^* mutant (Creface, C–D) XY gonads at E11.5. Dotted lines mark the gonads (A,B). Note the similar pattern of SRY expression for both genotypes.
(PDF)

Table S1 Primers sequences for real-time PCR.
(XLSX)
