# Supplementary figures and images for: Loss of Function Mutation in the Palmitoyl-Transferase HHAT Leads to Syndromic 46,XY Disorder of Sex Development by Impeding Hedgehog Protein Palmitoylation and Signaling
Source: PLoS Genet. 2014 May 1;10(5):e1004340. doi: 10.1371/journal.pgen.1004340 (PMC4006744; doi:10.1371/journal.pgen.1004340)

## SUPPLEMENTARY FIGURE 1

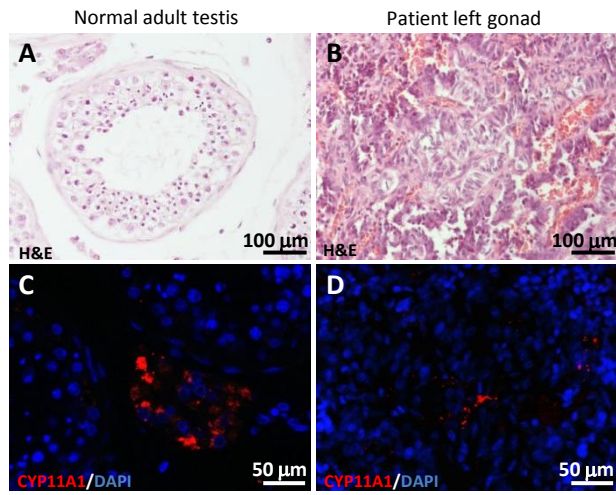

Supplement: Figure S1 — CYP11A1-expressing cells are reduced in the dysgenetic XY gonads of the patient with HHAT mutation. Haematoxylin and eosin staining (A,B) as well as immunostaining for the Leydig markers CYP11A1 (C,D) from testis of a fertile adult men (A,C) and the left gonads of the patient bearing G287V mutation in HHAT (B, D). (PDF) [file pgen.1004340.s001.pdf]

# SUPPLEMENTARY FIGURE 2

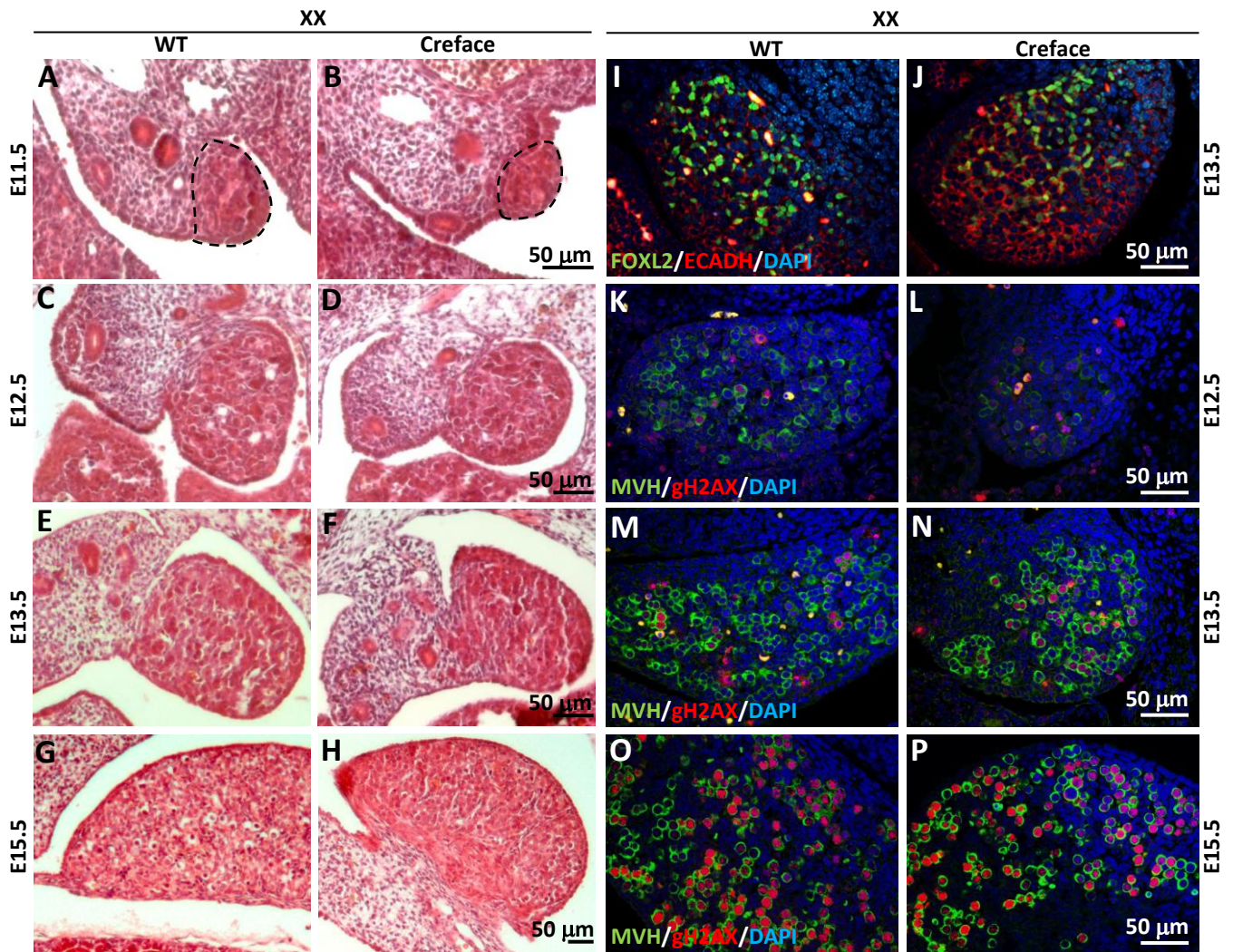

Supplement: Figure S2 — Ovarian differentiation is normal in XX mice embryos lacking Hhat. A–H) H&E stained sections of wild-type (WT) and HhatCreface/Creface mutant (Creface) XX gonads at E11.5 (A, B), E12.5 (C, D), E13.5 (E, F) and E15.5 (G, H). I–P) Expression of key ovarian markers was assessed by double immunofluorescence at E12.5, E13.5 and E15.5 on both wild-type (WT) and HhatCreface/Creface mutant (Creface) XX gonads using either FOXL2 (green) and the germ cell marker ECADH (red, I–J) or the meiotic marker γH2AX (red) along with the germ cell marker MVH (green; K–P). Dotted lines mark the developing gonads at E11.5 (A,B). (PDF) [file pgen.1004340.s002.pdf]

### SUPPLEMENTARY FIGURE 3

XY, E11.5

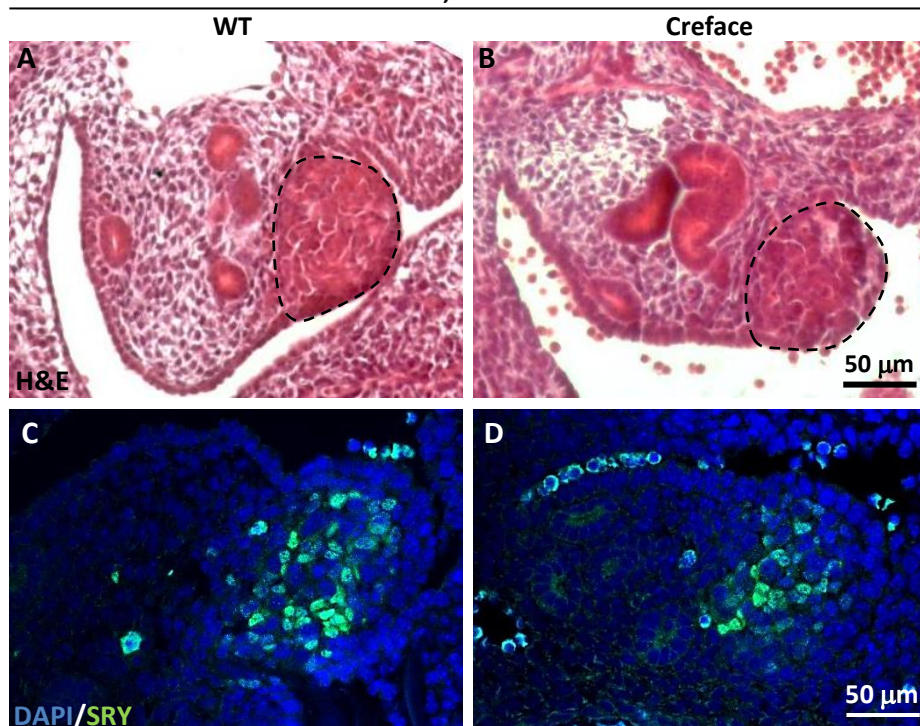

Supplement: Figure S3 — SRY is expressed in the genital ridges of XY embryos lacking Hhat gene. Haematoxylin and eosin staining (A,B) as well as immunostaining for SRY (C,D) from WT (A–C) and HhatCreface/Creface mutant (Creface, C–D) XY gonads at E11.5. Dotted lines mark the gonads (A,B). Note the similar pattern of SRY expression for both genotypes. (PDF) [file pgen.1004340.s003.pdf]
